# The Development and Validation of the SWADOC: A Study Protocol for a Multicenter Prospective Cohort Study

**DOI:** 10.3389/fneur.2021.662634

**Published:** 2021-04-29

**Authors:** Evelyne Mélotte, Marion Belorgeot, Roxanne Herr, Jessica Simon, Jean-François Kaux, Steven Laureys, Leandro R. D. Sanz, Aude Lagier, Dominique Morsomme, Frederic Pellas, Olivia Gosseries

**Affiliations:** ^1^Physical and Rehabilitation Medicine Department, University Hospital of Liège, Liège, Belgium; ^2^Coma Science Group, GIGA-Consciousness, University of Liège, Liège, Belgium; ^3^Centre du Cerveau, University Hospital of Liège, Liège, Belgium; ^4^Physical and Rehabilitation Medicine Department, University Hospital of Nîmes, Nîmes, France; ^5^Department of Speech and Language Pathology, Faculty of Medicine, University of Strasbourg, Strasbourg, France; ^6^Psychology and Neuroscience of Cognition, University of Liège, Liège, Belgium; ^7^Otorhinolaryngology Head and Neck Surgery Department, University Hospital of Liège, Liège, Belgium

**Keywords:** swallowing, dysphagia, consciousness, severe brain injury, assessment

## Abstract

**Background:** After a coma, patients with severe brain injury may present disorders of consciousness (DOC). A substantial proportion of these patients also suffer from severe dysphagia. Assessment of and therapy for swallowing disabilities of patients with DOC are essential because dysphagia has major functional consequences and comorbidities. Dysphagia evaluation in patients with DOC is impeded by the lack of adapted tools. The first aim of this study was to create a new tool, the SWallowing Assessment in Disorders Of Consciousness (SWADOC), and propose a validation protocol. The SWADOC was developed to help therapists assess factors related to swallowing in patients with DOC. The second aim was to investigate the relationship between patients' level of consciousness and SWADOC items and scores.

**Method/Design:** In this multicenter prospective cohort, 104 patients with DOC will be tested three times over five consecutive days with the SWADOC. Statistical analyses will focus on the reliability and validity of the SWADOC, especially the intrarater and interrater reliability, internal consistency, measures of dispersion, and concurrent validity with the Facial Oral Tract Therapy Swallowing Assessment of Saliva (FOTT-SAS). The level of consciousness will be assessed with the Simplified Evaluation of CONsciousness Disorders (SECONDs) and the Coma Recovery Scale-Revised (CRS-R).

**Discussion:** The assessment of swallowing abilities among patients with DOC is the first necessary step toward the development of a customized dysphagia care plan. A validated scoring tool will be essential for clinicians to better assess dysphagia in patients with DOC and document the evolution of their disorders.

**Trial Registration:** NCT04706689.

## Introduction

After a coma, some patients with severe brain injury will develop an altered state of consciousness before recovering partial or complete consciousness. Disorders of consciousness (DOC) consist of three states ranging from no awareness and no arousal to the preservation of arousal with fluctuating awareness (1): coma (2), vegetative state/unresponsive wakefulness syndrome (UWS) (3, 4), and minimally conscious state (MCS) (5). Patients with UWS typically exhibit only oromotor reflexes, blinks, and startle responses, as well as withdrawal from noxious stimuli (3). These patients do not respond to command and do not show visual pursuit or fixation. Individuals with MCS show reproducible but inconsistent signs of consciousness, such as following commands, visual pursuit or fixation, and localization of noxious stimuli (5). MCS has been subcategorized into MCS PLUS (MCS+) and MINUS (MCS–) based on the complexity of the observed responses: MCS+ describes high-level behavioral responses (i.e., following commands, intelligible verbalizations, intentional communication using a gestural or verbal yes/no code), and MCS– describes low-level behavioral responses (i.e., automatic motor behaviors, object manipulation, localizing objects in space, localizing noxious stimuli, visual pursuit or fixation) (6, 7). When patients recover the ability to functionally communicate or to use objects appropriately, we consider that they have emerged from MCS (EMCS) (5). The currently recommended scale for the behavioral assessment of consciousness is the Coma Recovery Scale–Revised (CRS-R) (8), as it fulfills all the Aspen Neurobehavioral Workgroup criteria (9). The CRS-R has an oromotor subscale that includes the assessment of basic oromotor reflexes and vocalizations or verbalizations, but no swallowing components are integrated in this scale. Recently, the Simplified Evaluation of CONsciousness Disorders (SECONDs) scale (10, 11) was developed based on the most prevalent signs of consciousness observed using the CRS-R (12). This tool is quick and easy to administer (11).

Almost all patients with DOC have severe dysphagia (13), requiring the use of ventilation and nutritional support (i.e., tracheostomy, gastrostomy) to limit the occurrence of comorbidities (e.g., bronchopulmonary infection, undernutrition). Individuals with DOC classically receive hydration and nutrition through an enteral feeding tube (14), and a large majority will not be able to return to exclusively oral feeding (13, 15). An objective swallowing assessment using fiber-optic endoscopic or videofluoroscopic swallowing evaluations is required in patients with DOC, for whom the possibility of partial or total oral feeding is being considered. These examinations, performed by experienced clinicians, constitute the gold standard tool for assessing dysphagia in this population. Indeed, these examinations allow clinicians to precisely analyze the mechanisms at play during swallowing and to detect possible silent aspirations, which are very prevalent in patients with DOC (13). As Mélotte et al. (13) showed in a cohort of 92 patients, the risk of silent aspiration is high for these patients, as 48% of DOC patients do not have a cough reflex. Moreover, 28% presented saliva aspiration, 13% aspiration with thick texture, and 32% aspiration with liquid texture. A lack of knowledge of the specific features of DOC prevents some clinicians from performing these exams. Moreover, performing a functional swallowing test (liquid and food test) can be difficult in patients with severe trismus (lockjaw) or total absence of the oral phase of swallowing, but as long as a partial oral phase with initiation of swallowing exists, thick or liquid swallowing can be tested (13, 16–18).

In daily practice, speech therapists help document the presence or absence of a range of components required for swallowing in order to guide therapy and monitor its effectiveness by repeated assessment over time. The clinical reality of patients with DOC, mainly the lack of response to simple motor commands (in UWS and MCS–) and functional communication, makes it difficult to fully understand and appropriately treat their swallowing disorders. For these reasons, classical swallowing assessment at the bedside can be unsuitable for this population. There is a lack of appropriate bedside tools to appraise and monitor swallowing disorders in patients with DOC. The impossibility of orienting interventions based on determined quantitative and qualitative swallowing components makes it more difficult to develop a treatment plan and assess the patient's progress.

Our recent DOC cohort study (13) found some links between swallowing and the level of consciousness. In particular, none of the patients with UWS and only a minority of the patients in MCS exhibited an effective oral phase of swallowing (adequate lip prehension, tongue propulsion, and no post-swallowing oral stasis). However, these links are not yet completely understood, and further studies are necessary to increase our knowledge of which components of swallowing are linked to consciousness and to what extent. In this context, the validation of a tool that focuses on qualitative and quantitative swallowing components will help us identify swallowing behaviors that may be considered unequivocal signs of consciousness in patients with DOC. These results may eventually contribute to the development of new diagnostic guidelines for DOC that would include swallowing behaviors in their criteria.

The aim of this article is to present a protocol that develops and validates the SWallowing Assessment in Disorders Of Consciousness (SWADOC). This tool will be administered repeatedly to a population of patients with DOC by different examiners, and intrinsic test characteristics will be calculated. The relationship between patients' level of consciousness and SWADOC scores will also be studied.

## Methods And Analysis

### Development of the Swallowing Assessment in Disorders of Consciousness

#### Identification of Domain(s) and Item Generation

The SWADOC was developed to overcome the lack of a suitable tool that would allow clinicians to assess and measure swallowing-related components in patients with DOC. It explores some of the oral and pharyngeal components of swallowing as well as a range of prerequisites and related components of swallowing. Our first aim was to develop a rapid, reliable quantitative tool. However, although quantitative items present advantages, many qualitative elements are also meaningful in fully understanding a patient's profile. Thus, we decided to include both quantitative and qualitative items.

This tool was developed by three speech therapists and then submitted for evaluation to 10 experts (otorhinolaryngologists and speech therapists) who work with patients with DOC. Their comments contributed to the final version. The development period lasted ~10 months.

The tool was developed based on both deductive and inductive methods (19). First, we examined literature in the field of consciousness and swallowing, and looked for existing scales (deductive method). The construction of the tool was inspired by actual knowledge on dysphagia in DOC patients based on the few studies dedicated to swallowing in DOC patients (13, 15, 18, 20).

The SWADOC was built based upon several existing tools: the Facial Oral Tract Therapy (FOTT) (21, 22), the SECONDs (10, 11), the New Zealand Secretions Scale (NZSS) (23), the Oral/Facial Sensitivity subtest of the Comprehensive Assessment Measure for Minimally Responsive Individuals (CAMMRI) (24), the stimulation method for sensory processing disorder (sensory dysorality) proposed by Senez (25), and the arousal protocol of the CRS-R (8). It includes some medical history information from the Food Intake LEVEL Scale (FILS) (26) and the International Dysphagia Diet Standardization Initiative (IDDSI) (27).

The SWADOC was inspired by the FOTT (22), as adapted for patients with DOC in French by Bicego et al. (21), and modified based on our day-to-day clinical experience with patients with DOC. The items assessing command following are based on the SECONDs principles (10, 11), insofar as response to verbal command is considered intact if the patient passes a minimum of two out of three trials. The item on quantitative saliva secretions is based on the NZSS (21), which assesses secretion severity during endoscopy by location, amount (as a percentage), and response. We used the percentages proposed in that scale and adapted them to the oral cavity. Since the oral area is larger than the laryngeal area, the percentages are more difficult to evaluate, but to our knowledge, there is no validated scale for the evaluation of accumulated secretions in the mouth. Tactile stimulation is inspired by Senez's stimulation method for sensory processing disorder (25). Finally, the SWADOC requires the examiner to perform an arousal protocol similar to the CRS-R (8) if the patient falls asleep during the procedure. The medical history taking subsection also features the IDDSI (27) score and the FILS (26) score.

We then applied the tool in routine clinical practice with patients with different levels of consciousness and adjusted some items as a result (inductive method).

#### Content Validity

Ten experts were asked to judge the SWADOC overall and per item with a Likert scale to solicit their opinion of its relevance in their clinical practice, its suitability for patients with DOC, and the clarity of the administration guidelines. The experts' responses focused on the clarity of certain instructions and suggested that some qualitative items be added and some quantitative items modified (levels that were too subjective or dependent on hospital functioning and not on patients themselves). These suggestions were analyzed and taken into account to improve the SWADOC by clarifying instructions, adding qualitative items, and modifying quantitative items.

### Presentation of the Tool

**Figure 1:**
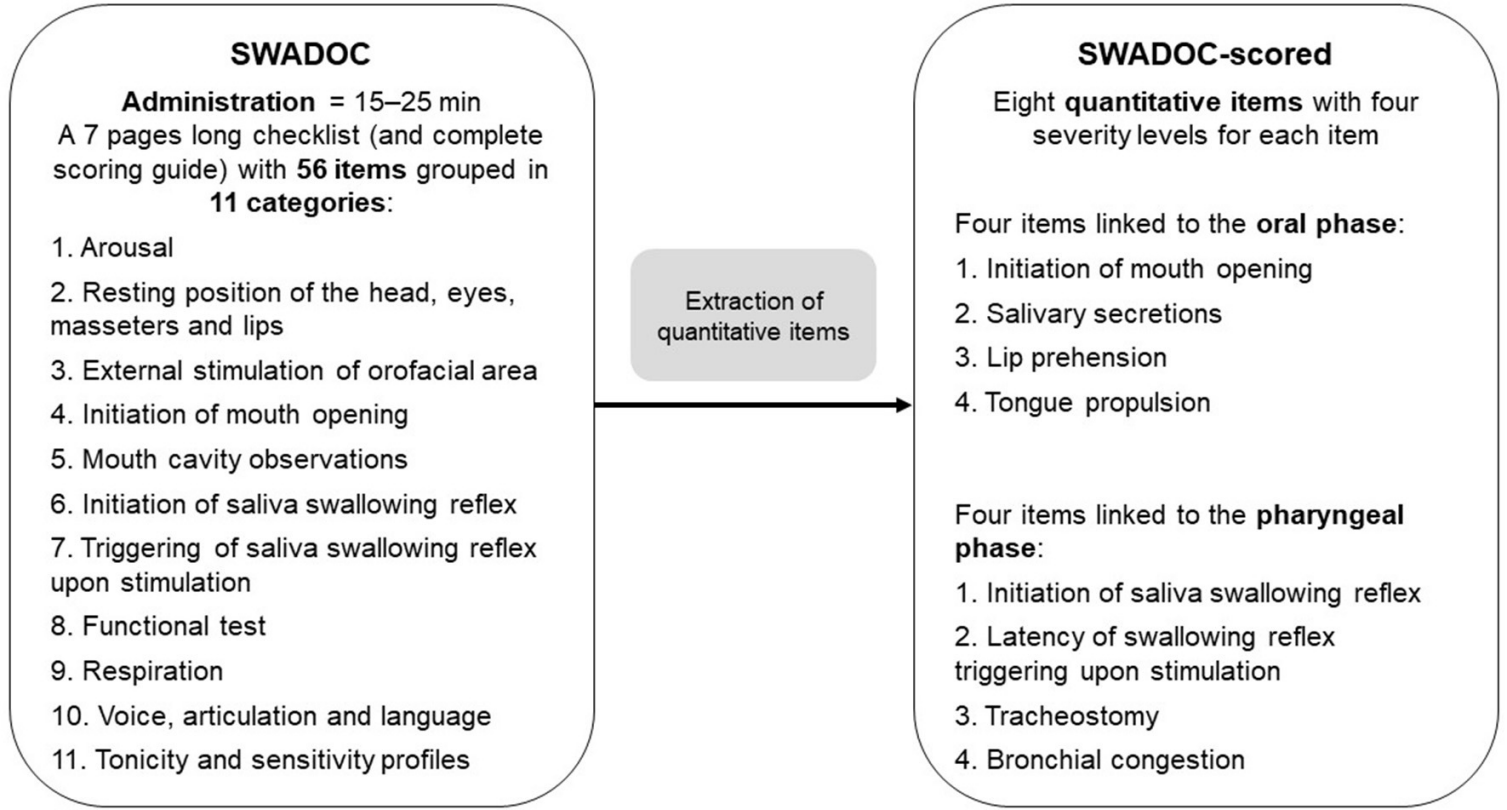
Description of the SWADOC. ENT, Ear, Nose, and Throat; SWADOC, SWallowing Assessment in Disorders Of Consciousness.

The tool is composed of 56 items: 48 qualitative items and a subsection called the “SWADOC-scored” comprising eight quantitative items. The instruction guide comprises (1) one page with general recommendations; (2) a list of required materials; (3) explanations of the quantitative items; (4) a detailed medical history; (5) the SWADOC-scored grid; and (6) a checklist and seven pages of instructions covering all the quantitative and qualitative items (Figure 1; Supplementary Materials 1, 2). The tool was translated into English for the convenience of readers of this paper. However, if an English validation is carried out, a back-translation method will be applied.

**Figure 2:**
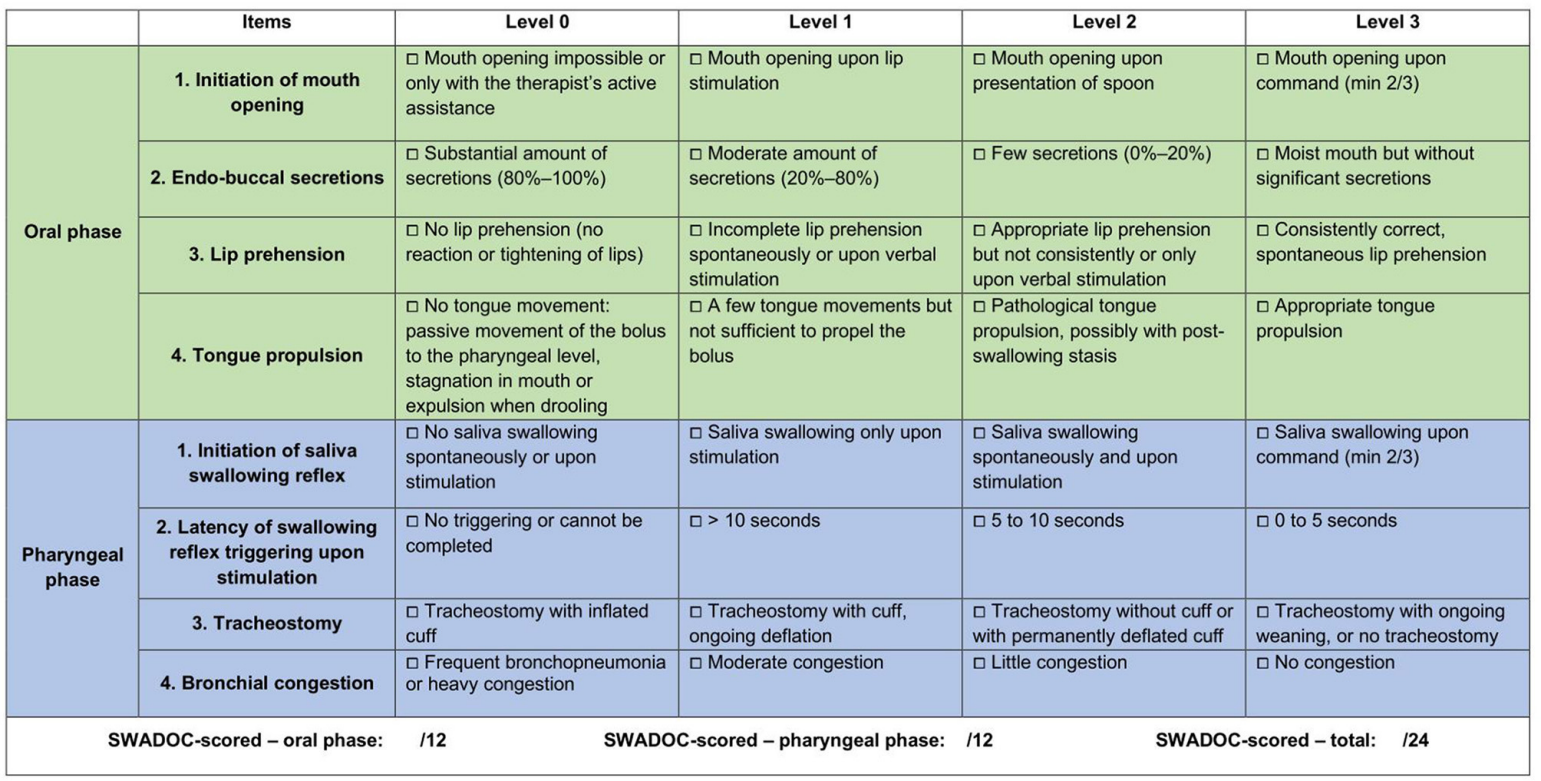
SWADOC-scored (English version). DOC, disorders of consciousness; SWADOC, SWallowing Assessment in Disorders Of Consciousness.

The “SWADOC-scored” subsection includes eight quantitative items (Figure 2; Supplementary Material 3). Four items are linked to the oral phase and four to the pharyngeal phase. For each quantitative item, patient's abilities are rated on a four-level scale ranging from 0 to 3. These levels correspond to item scores that can be added together to calculate three performance scores: the oral phase subscore (sum of the four oral item scores), the pharyngeal phase subscore (sum of the four pharyngeal item scores), and the total swallowing score (sum of the eight item scores).

The administration procedure for the test and the sequencing of items were designed to put patients in optimal conditions for the exam. For example, the therapist first introduces himself/herself, describes the assessment procedure, and then begins with external facial stimulation before doing the mouth cavity stimulation. The therapist assesses the patient at the bedside or in his or her usual chair and only if the patient is awake and shows no signs of pain or medical problem (e.g., fever, hypoxemia, and arrhythmia). In addition, during the assessment, some parameters for stopping are provided to avoid presenting stimulations that are inappropriate for the patient's abilities (e.g., swallowing a minimal amount of thickened liquid). All these recommendations are described in the administration guide.

The objectives of the SWADOC are to address all of the goals of an assessment tool: (1) document the prerequisites for swallowing; (2) determine the active ingredients of the therapy; (3) track changes in a patient's swallowing abilities; and (4) monitor the effectiveness of a therapy. In a scientific research context, the SWADOC will help us better understand the links between the level of consciousness and swallowing components.

### Study Design

This project is a multicenter prospective cohort study that will take place in several hospitals and clinics in Belgium and France. The SWADOC was created in French.

### Population and Recruitment

This study will be carried out in patients with DOC or emerging from DOC following severe acquired brain injury. Patients will be divided into four groups according to their clinical diagnosis, as assessed with the SECONDs and with the CRS-R: UWS, MCS–, MCS+, and EMCS. Participants will be recruited from inpatient neurological rehabilitation programs in post-coma units and rehabilitation services or among patients hospitalized for a multimodal assessment of consciousness for diagnostic and prognostic purposes.

Only patients who meet all of the following inclusion criteria will be considered for enrollment: (1) age above 18 years; (2) perfect knowledge of French language before the injury; (3) previous coma phase caused by a severe acquired brain injury; (4) medical stability (no mechanical ventilation or sedation, no acute medical pathology such as infection or respiratory distress); (5) no neurological or otorhinolaryngological disease that can impact swallowing prior to the brain injury; (6) minimum of 28 days since the acquired brain injury at inclusion (28, 29); (7) diagnosis of UWS, MCS–, MCS+, or EMCS based on the SECONDs and the CRS-R; (8) informed consent from the patient's legal representative; and (9) affiliated patient or beneficiary of a health insurance plan (for participants in France only).

### Validation of the Study Procedure

Each patient enrolled in the validation study will have his or her level of consciousness assessed at baseline with a single administration of the CRS-R. Next, the level of consciousness and swallowing abilities will be assessed with three tools during three separate sessions (only one administration of the FOTT-SAS) in the morning or the afternoon in order to assess the tool's intrarater and interrater reliability. The baseline assessment and sessions will be spread over 5 consecutive days.

1. CRS-R: This is a standardized neurobehavioral assessment of consciousness composed of six subscales (auditory, visual, motor, oromotor/verbal, communication, and arousal). It assesses different functions with various numbers of hierarchically arranged items that distinguish UWS, MCS, and EMCS patients. The total score is composed of the sum of the maximum scores obtained in each subscale.
2. SECONDs: It is shorter to administer (median of 7 min) and requires only a mirror as material. It consists of eight items: observation and reporting of spontaneous behaviors, response to command, communication, visual pursuit and fixation, pain localization, oriented behaviors, and arousal. We have chosen this consciousness tool during sessions rather than the CRS-R because of its short duration so that the SWADOC can be administered immediately before or after, given that attentional abilities are often reduced in patients with DOC. The SECONDs provides a total score directly reflecting one diagnosis (0 = coma; 1 = UWS; 2–5 = MCS–; 6–7 = MCS+; 8 = EMCS).
3. SWADOC: This tool includes a battery of 56 quantitative and qualitative items. Quantitative items are grouped in the SWADOC-scored, with oral and pharyngeal subscores and a total score. Running the whole SWADOC lasts between 15 and 25 min based on preliminary tests.
4. FOTT-SAS: The results of the SWADOC-scored in one session will be compared to those of the FOTT Swallowing Assessment of Saliva (FOTT-SAS) (30). The test comprises seven questions; if items 1–4 are answered “Yes” and items 5–7 are answered “No,” oral intake should be initiated (Supplementary Material 4). The FOTT-SAS includes items that can be scored based on the administration of the SWADOC. In that respect, no additional administration will be required.

**Figure 3:**
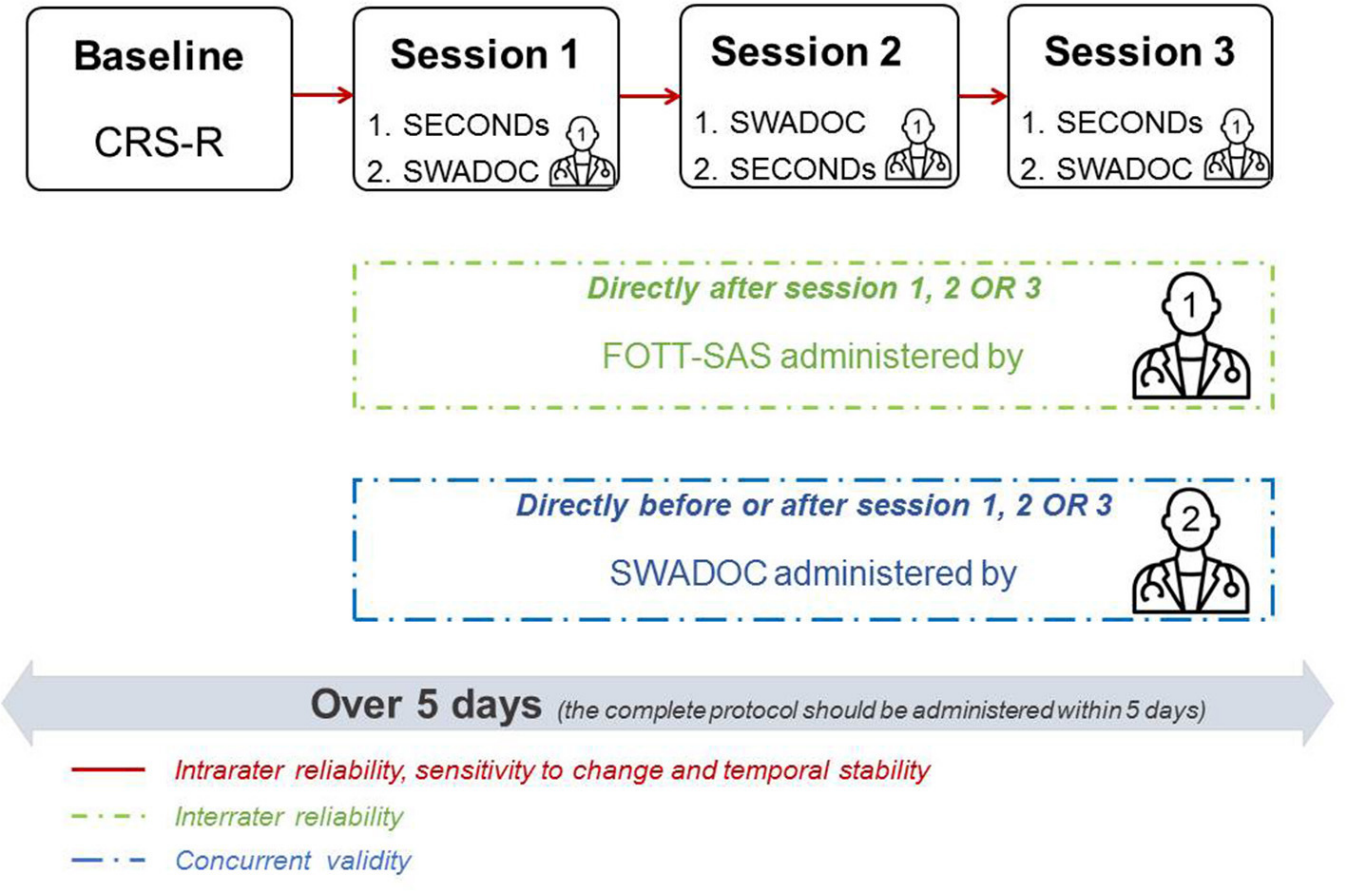
Study protocol. Example of the assessment program for a patient. The program for each patient is organized such that the three sessions take place over 5 days in the morning or in the afternoon. The SECONDs is administered before or after the SWADOC. Examiner 2 will administer the SWADOC before or after examiner 1 in one of the three sessions. Examiner 1 will score the FOTT-SAS after one of the three sessions. SECONDs, Simplified Evaluation of CONsciousness Disorders; SWADOC, SWallowing Assessment in Disorders Of Consciousness; FOTT-SAS, Facial Oral Tract Therapy Swallowing Assessment of Saliva.

The clinical protocol procedure is illustrated in Figure 3.

Because of clinical realities, no attempt will be made beforehand to standardize the order (SECONDs before or after SWADOC and examiner 2 before or after examiner 1) or time of day (morning or afternoon) of evaluations. However, during data collection, efforts will progressively be made to balance the order and time of day and the time of evaluation of examiner 2 (before or after examiner 1).

The administration of the SWADOC at each session will allow us to assess the tool's intrarater reliability (temporal stability). Directly before or after one of the three sessions, a second examiner will administer the SWADOC a second time to test its interrater reliability. The second assessment will be blinded to the results of the first. To be able to take into account potential mismatches between the ratings of the two examiners, each scoring protocol will be analyzed directly after the interrater session and a discussion will follow to try to understand the reasons for any discrepancies. This information will be noted in the protocol. After the recruitment procedure and depending on the degree of agreement based on the statistical analysis, modifications of the instructions, and/or items will be considered based on the qualitative information collected from the examiners.

Sensitivity to change will determine whether changes in the level of consciousness (variations in SECONDs scores) result in changes in the SWADOC-scored across the three assessments of the same patient. Repeating the SECONDs assessment will also allow for a reliable assessment of the patient's level of consciousness (UWS, MCS–, MCS+, or EMCS) and decrease the risk of misdiagnosis (31).

### Study Hypotheses

Our main hypothesis is that the SWADOC is reliable and valid.

Our second hypothesis is that the subscores (oral phase and pharyngeal phase) and the total score are related to the patient's level of consciousness. Moreover, based on previous studies (13, 15), we expect that UWS patients will be at level 0 for oral phase items 1, 3, and 4 of the SWADOC-scored: no mouth opening at spoon approach, no spontaneous lip prehension, and no appropriate tongue propulsion.

### Data Analysis

The statistical significance will be set at *p* < 0.05.

#### Power of the Study

The sample size was calculated using a power calculation (G * Power, Universities of Kiel, Mannheim, and Düsseldorf) in a one-way analysis of variance (ANOVA) with a significance level of 0.05 and a power of 0.8. Effect size is based on previous published data on swallowing performance in patients with DOC (13). Cohen's *d* was used to estimate the effect size; the result was 0.659, corresponding to an eta-squared of 0.0979. We round up and base our calculation on an effect size of 0.1, corresponding to an intermediate effect. In this context, a total sample size of 104 participants (26 per group) is needed to demonstrate a difference in SWADOC‘ subscores and total score between the four consciousness groups. If, by the end of June 2025 the sample size is not reached, we will discuss extending the data collection period if necessary.

#### Descriptive Analysis

First, descriptive statistics will be performed to describe the entire group and each diagnostic group (UWS, MCS–, MCS+, and EMCS) for age, gender, etiology, and time since insult. To test gender independence between groups, we will use chi-square tests. For the other variables, we will perform a one-way ANOVA with group as an independent variable. Homogeneity of variance will be assessed using Levene's test. In case of violation, we will use Welch's approximation of a ANOVA.

#### Reliability

The intrarater (i.e., SWADOC-scored vs. SWADOC-scored by the same examiner on the same day and on two different days) and interrater reliability (i.e., SWADOC-scored vs. SWADOC-scored on the same day by two different examiners) will be calculated in two separate analyses using weighted Fleiss's kappa coefficients (K_W_). A value below 0 will be considered to indicate poor agreement, between 0 and 0.2 slight agreement, between 0.21 and 0.4 fair agreement, between 0.41 and 0.6 moderate agreement, between 0.61 and 0.8 substantial agreement, and between 0.81 and 1 almost perfect to perfect agreement (32). We will also determine the internal consistency of the SWADOC-scored with Cronbach and Spearman intercorrelations to determine the interrelatedness of the constituent items.

#### Validity

##### Concurrent Validity

The results of the SWADOC-scored will be compared to the FOTT-SAS score for the same time and with the best score using Pearson's correlation (parametric test) or Kendall's correlations (nonparametric test) after examination of the data distributions.

##### Measures of Dispersion

The distribution of total SWADOC-scored scores will be examined to determine whether performance on the scale is evenly distributed across the range of possible scores, and each item will be analyzed to identify possible floor or ceiling effects. Based on these results, we will consider the need to modify the scale accordingly.

#### Relationship Between Swallowing Components and Levels of Consciousness

The differences in SWADOC-scored items, oral and pharyngeal subscores for the SWADOC-scored, and total score for the SWADOC-scored for the consciousness diagnostic groups will be assessed using a comparison of means with a one-way ANOVA. Violation of the homogeneity of variance will be checked using Levene's test. If this is the case, we will use Welch's approximation instead of an ANOVA. If there is a significant main effect, we will perform Tukey's honestly significant difference (HSD) test to compare all pairwise differences. If there is a severe violation of the normality, we will perform a Kruskal–Wallis test with the Dwass-Steel-Critchlow-Fligner (DSCF) multiple comparison analysis. Partial eta-squared will be used as a measure of effect size. *Post-hoc* multiple comparisons will be conducted to identify where significant differences between consciousness groups exist. SWADOC scores will be the dependent variable, while consciousness group will be the independent variable. Statistical analysis will be performed by a researcher blind to the consciousness category.

## Discussion

It is challenging to assess swallowing in patients with DOC because of the clinical reality of this population. This protocol describes a clinical study that will seek to validate the SWADOC, a tool adapted for bedside swallowing assessments in patients with DOC. The link between patients' level of consciousness and SWADOC scores will also be studied. To achieve these goals, a multicenter prospective cohort study design will be adopted.

### Strengths and Opportunities

To our knowledge, this is the only published study protocol that seeks to validate a swallowing assessment tool for patients with DOC. To meet all the criteria for an optimal assessment tool (i.e., appraise patients' abilities, monitor their progress, measure the effect of a given therapy, allow comparison with other patients), the SWADOC is composed of both quantitative and qualitative items.

The quantitative items will help measure any changes and treatment effects, while the qualitative items will help clinicians provide a clear and accurate summary of the patient's strengths and weaknesses, and thus orient his or her therapy in the best possible way. Depending on the treatment plan, the dysphagia therapy may be oriented more toward active stimulation to improve salivary control and efficiency of the oral and pharyngeal phases or toward maximizing the patient's comfort.

In a second phase, it would be interesting to compare the results of the SWADOC with an objective assessment (fiber-optic endoscopic evaluation of swallowing or videofluoroscopic swallowing study) to determine its predictive validity and investigate its construct validity. However, the SWADOC is not intended to be the only tool used to determine the possibility of reintroducing oral nutrition for the patient: we acknowledge the need to objectively evaluate patients with DOC before reintroducing oral feeding, as the risk of inhalations and silent aspirations is very high. The SWADOC will complement the therapeutic framework for dysphagia among patients with DOC in order to set up the active components of therapy (33), measure the efficacy of the therapy, and orient therapists toward an objective examination when the patient's recovery seems to allow it.

### Limitations and Pitfalls

There are a number of limitations on this study especially because of the clinical challenges of dealing with patients with DOC. The main limitation is the feasibility of having each patient undergo a baseline administration of the CRS-R and three assessments. This group's arousal fluctuates considerably, as they are highly sensitive to stress and fatigue, and often subject to medical complications (e.g., vomiting, pain, respiratory infections, etc.) that may limit their availability to participate in the study. To take into account this pitfall of the application of the methodology to patients with DOC, we chose to extend the evaluation period to five days rather than two days as initially planned.

Although, we have developed the most accurate levels possible for each item and a complete scoring guide, the SWADOC is based on subjective observations of swallowing components. The interrater reliability will help us determine the consistency of the rating system between examiners. Depending on these results, the scale may be adapted.

Furthermore, the decision to assess the level of consciousness using the SECONDs may seem questionable because this scale is recent, but it is derived from the CRS-R, which is known to be the gold standard scale for behavioral assessment of consciousness. Nevertheless, the SECONDs is much quicker to administer and includes the five CRS-R items that detect 99% of MCS patients (12). Moreover, this scale has good intra- and inter-rater reliability (kappas ranging from 0.85 to 0.91 and 0.82 to 0.85, respectively). However, we considered adding one administration of the CRS-R at baseline before the three sessions because the CRS-R gives more details and precision on the patient's overall state, including reflexes.

## Ethics Statement

This research protocol was reviewed and approved by the two central ethics committees (Ethics Committee of the Faculty of Medicine of the University Hospital of Liège (2020-79) and Ethics Committee of Île de France XI (20.05.26.70621), as well as by the Ethics Committees of the participating hospitals and clinics. The patients' legal guardians/participants provided their written informed consent to participate in this study.

## Dissemination

Information documents and explanations of the study will be given to the patients' legal representatives to give them all the necessary information to make an informed decision about participation in the study. Written informed consent, including the name and contact information of the investigators in charge of the study, will be obtained from all patients' legal representatives prior to participation. The information form will also contain a paragraph indicating that the investigators in charge of the study have insurance that will cover accidental damages. The legal representatives will be informed that they can choose not to participate in the study without any consequences for the patients' quality of care and may, at any time and without giving a reason, withdraw from the study. All information collected during this study will be kept confidential. The data will be pseudo-anonymized and listed under an ID code accessible only to the researchers in charge of the study and protected by a firewall. The principal investigators of the study (EM, MB, and OG) are responsible for these data. Data management will comply with the General Data Protection Regulation (EU 2016/679) including the fact that data will be used during scientific presentations and publications without mentioning the participants' identity.

## Author Contributions

EM, MB, and RH developed the SWADOC. EM, MB, RH, and OG contributed to the conception and design of the study and drafted the manuscript. JS, J-FK, AL, DM, LRDS, SL, and FP made major revisions of significant portions of the manuscript. FP and OG contributed equally. All authors contributed to the article and approved the submitted version.

## Conflict of Interest

The authors declare that the research was conducted in the absence of any commercial or financial relationships that could be construed as a potential conflict of interest.

## Abbreviations

CAMMRI: Comprehensive Assessment Measure for Minimally Responsive Individuals
CRS-R: Coma Recovery Scale–Revised
DOC: Disorders Of Consciousness
EMCS: Emergence from the Minimally Conscious State
FILS: Food Intake LEVEL Scale
FOTT: Facial Oral Tract Therapy
FOTT-SAS: Facial Oral Tract Therapy Swallowing Assessment of Saliva
IDDSI: International Dysphagia Diet Standardization Initiative
MCS: Minimally Conscious State
NZSS: New Zealand Secretions Scale
SECONDs: Simplified Evaluation of CONsciousness Disorders
SWADOC: SWallowing Assessment in Disorders Of Consciousness
UWS: Unresponsive Wakefulness Syndrome.
