# Supplementary material for: The Development and Validation of the SWADOC: A Study Protocol for a Multicenter Prospective Cohort Study
Source: Front Neurol. 2021 Apr 29;12:662634. doi: 10.3389/fneur.2021.662634 (PMC8116670; doi:10.3389/fneur.2021.662634)
Supplement: Supplementary file 1 [file Presentation_1.pdf]

Supplementary Material 1. English version of the SWADOC's Administration Guide

Note: Non-validated translation for understanding only

Administration Guide

The SWADOC is a swallowing assessment protocol adapted for patients with disorders of consciousness (DOC). It was designed because of the lack of tools to assess swallowing-related components that were adapted to the specific features of patients with DOC (no response to commands in some cases and inability to communicate functionally). The purpose of the SWADOC is to explore certain components related to the oral and pharyngeal phases of swallowing and a series of prerequisites and other components related to swallowing. It cannot be the sole basis for judging the possibility of resuming eating, in whole or in part. In our opinion, such a decision regarding patients with DOC can only be made after an objective examination of swallowing (videofluoroscopic or nasofibroscopic examination).

The tool is non-invasive and can be used in clinical practice at patients' bedside, as an initial assessment and then repeatedly to track patients' progress.

This protocol can be used by clinicians working with patients with DOC, as a basis for both quantitative and qualitative analysis of their swallowing, which is useful in formulating their treatment plan. In addition, the quantitative items (SWADOC-scored) allow one to compute a total score and subscores for the oral and pharyngeal phases of swallowing, which can constitute a baseline for assessing the efficacy of a treatment for the patient.

This guide includes: (1) some general information to be read prior to the assessment; (2) a list of the necessary materials; (3) the items to be checked during the medical history taking; (4) specific information concerning the scoring of quantitative items; and (5) the SWADOC-scored grid (only quantitative items). You will then find a table with instructions for administering and scoring each item, both qualitative and quantitative. Finally, the appendices contain further information on the tests or protocols cited and bibliographic references.

1. General comments

- It is preferable for the patient to be in a sitting position during the assessment.
- While the test is being administered, the therapist must inform the patient about each stimulation to be administered.
- Before starting the patient's assessment, it is important to be sure that none of the following factors applies. If one of them does, the assessment should be delayed. Similarly, if one of these factors arises during the assessment, the assessment must be stopped: fever, ongoing infection, oxygen desaturation, unstable heart rate, autonomic crisis, and inability to keep the patient awake (a maximum of 3 arousal protocols may be applied during an assessment).

2. Materials needed for the assessment

- 2 stopwatches 🕓
- Flashlight
- Teaspoon
- Cotton swab soaked in a cold, sweet solution
- 5 mL of cold, colored thickened solution with IDDSI level 3 texture (moderately thick)[1]

3. Quantitative items

- Level 3 is the expected standard. The higher the total score, the better preserved the patient's swallowing ability is.
- A patient at time "T" cannot be at several different levels for the same item. If a patient responds at several levels, score him/her at the highest one (e.g., if a patient is able to open his/her mouth when the spoon approaches – level 2 – and on command – level 3 – the score will be level 3).
- To calculate the total score and subscores: assign 3 points for every level 3 item, 2 points for every level 2 item, 1 point for every level 1 item, and 0 points for every level 0 item. Add the scores for all items in the oral phase to calculate the oral subscore, add the scores for all items in the pharyngeal phase to calculate the pharyngeal subscore, and add the oral and pharyngeal subscores to calculate the total score.

4. Medical history

The information required for the medical history should be collected from the patient's file or from the care team.

**Date and time of administration:**<br>**Patient's last and first names:**<br>**Sex:**<br>**Date of birth:**<br>**Current residence:**<br>**Receiving speech therapy:** yes / no<br>- **Frequency:**<br>- **Place:** at a center / at home<br>- **Type:**	**Nutritional status:**<br>- Current weight:<br>- BMI (weight/height²):<br>- Weight loss or gain during the last month: …........... – during the last 6 months: …....<br><br>**FILS score**[2] *(see Appendix 1)* and corresponding level: …………<br>For a FILS score of less than 7, specify the **enteral feeding method:**<br>◻ gastrostomy ◻ jejunostomy ◻ nasogastric tube<br>For a FILS score of more than 3, specify the **IDDSI score**[1] (or equivalent – *see Appendix 2*) **for foods:** ……….

**Date of accident:**<br>**Type of accident:**<br>☐ Vascular<br>☐ Traumatic<br>☐ Anoxic<br>☐ Other:<br><br>**Location of brain lesion:**<br><br>**Glasgow score at time of accident:**<br>- Eye opening:<br>- Verbal response:<br>- Motor response:	**Help required during meals:** yes / no – if yes, from whom: ………………………….<br>**Hydration:** ◻ enteral ◻ oral<br>In case of oral hydration, specify the **IDDSI score**[1] **for drinks:**<br><br>**ENT problems before accident:** yes / no – specify:<br>…………………………………………………………………<br>**ENT problems since accident:** yes / no – specify:<br>…………………………………………………………………<br>**Ongoing or recent lung infections:** yes / no – specify:<br>…………………………………………………………………<br><br>**Respiratory kinesiotherapy:** yes / no – if yes, specify the frequency of sessions:<br>…………………………………………………………………<br><br>**Medication that may affect:** ◻ consciousness ◻ swallowing ◻ salivation – specify:<br>…………………………………………………………………<br><br>**Hypersalivation:** yes / no – **Treatment of hypersalivation:** ◻ toxins ◻ patches<br><br>Patient able to produce: ◻ spontaneous sounds ◻ words ◻ reliable communication<br>**Communication code:** yes / no – specify:<br>…………………………………………………………………<br><br>**Scores on the SECONDs**[3,4] **or the CRS-R**[5] at the time of assessment and corresponding level:<br>……………………………………….

5. Presentation grid for quantitative items (SWADOC-scored)

	Items	Level 0	Level 1	Level 2	Level 3
**Oral phase**	**1. Initiation of mouth opening**	◻ Mouth opening impossible or only with the therapist's active assistance	◻ Mouth opening upon lip stimulation	◻ Mouth opening upon presentation of spoon	◻ Mouth opening upon command (min 2/3)
	**2. Endo-buccal secretions**	◻ Substantial amount of secretions (80%–100%)	◻ Moderate amount of secretions (20%–80%)	◻ Few secretions (0%–20%)	◻ Moist mouth but without significant secretions
	**3. Lip prehension**	◻ No lip prehension (no reaction or tightening of lips)	◻ Incomplete lip prehension spontaneously or upon verbal stimulation	◻ Appropriate lip prehension but not consistently or only upon verbal stimulation	◻ Consistently correct, spontaneous lip prehension
	**4. Tongue propulsion**	◻ No tongue movement: passive movement of the bolus to the pharyngeal level, stagnation in mouth or expulsion when drooling	◻ A few tongue movements but not sufficient to propel the bolus	◻ Pathological tongue propulsion, possibly with post-swallowing stasis	◻ Appropriate tongue propulsion
**Pharyngeal phase**	**1. Initiation of saliva swallowing reflex**	◻ No saliva swallowing spontaneously or upon stimulation	◻ Saliva swallowing only upon stimulation	◻ Saliva swallowing spontaneously and upon stimulation	◻ Saliva swallowing upon command (min 2/3)
	**2. Latency of swallowing reflex triggering upon stimulation**	◻ No triggering or cannot be completed	◻ > 10 seconds	◻ 5 to 10 seconds	◻ 0 to 5 seconds
	**3. Tracheostomy**	◻ Tracheostomy with inflated cuff	◻ Tracheostomy with cuff, ongoing deflation	◻ Tracheostomy without cuff or with permanently deflated cuff	◻ Tracheostomy with ongoing weaning, or no tracheostomy
	**4. Bronchial congestion**	◻ Frequent bronchopneumonia or heavy congestion	◻ Moderate congestion	◻ Little congestion	◻ No congestion
**SWADOC-scored – oral phase: /12**		**SWADOC-scored – pharyngeal phase: /12**		**SWADOC-scored – total: /24**	

6. Protocol for assessing swallowing in patients with disorders of consciousness

Procedure for examination	Instructions	Scoring (qualitative items – quantitative items oral phase – quantitative items pharyngeal phase)	Materials
**1. Arousal**	When you enter the room, check the patient's arousal:<br>→ If the patient is awake, introduce yourself and start the assessment: **Greet the patient** orally, using his/her first name and touching his/her hand or arm. **Introduce yourself** and explain why you are there: for example, "Hello, my name is… I'm a speech therapist. I've come to see how you're doing and suggest some stimulation activities and exercises for you."<br>→ If the patient is asleep, try to wake him/her by using any kind of sensory stimulation: changing his/her position, increasing the light in the room, touching or speaking to him/her, etc. If this does not work, use the CRS-R[6] arousal protocol.<br><br>At the end of the assessment, note how long the patient kept his/her eyes open.	**Waking time:** ◻ 0%–25% ◻ 25%–50% ◻ 50%–75% ◻ 75%–100%<br><br>**Stimulation needed:** ◻ Yes ◻ No<br>*If yes:*<br>◻ Auditory – specify:<br>……………………………………………………………………<br>◻ Tactile – specify:<br>……………………………………………………………………<br>◻ Visual – specify:<br>……………………………………………………………………	
**2. Resting position of the head, eyes, masseters and lips**	**Resting position:** Note the position of the head, lips and masseters at rest, whether there is any facial or lip paralysis, and whether the eyes are open.<br><br>If members of the care team or the family are present, ask whether this is the "usual" head position.<br><br>Position yourself on the side where the patient's gaze is oriented, based on the head position.	→ **Patient's position:** ◻ in bed ◻ in (wheel)chair<br>→ **Resting head position:**<br>- *Verticality:* ◻ Neutral ◻ Flexion ◻ Extension<br>- *Horizontality:* ◻ Neutral ◻ R rotation ◻ L rotation<br>◻ R tilt ◻ L tilt<br>→ **Lips at rest:**<br>◻ Normal contact ◻ No contact<br>◻ Hypertonic contact (tightening)<br>→ **Masseter tone at rest:**<br>◻ Relaxed (normal) ◻ Hypertonia<br>◻ Hypotonia ◻ Contracted-relaxed<br>→ **Paresis, if any:** ◻ facial ◻ labial ◻ none<br>+ *side:* ◻ left ◻ right<br>→ **Eyes open:**<br>◻ Both eyes<br>◻ Possible to open both eyes but one eye open preferentially: ◻ L ◻ R<br>◻ Only possible to open one eye<br>→ **Spontaneous head movements:**<br>◻ yes ◻ no<br>→ **Resting respiration:** ◻ mouth ◻ nose ◻ tracheostomy	

**3. External stimulation of orofacial area**	→ **Passive head mobilization:** Place an open hand at the base of the skull and try to lightly move the head in different directions.<br>→ **Head support:** Remove the cushion or lift the head if it is flexed and note whether the head can be supported for the next 10 seconds.	→ **Passive head mobility:**<br>☐ Possible without resistance<br>☐ Possible but with resistance and tension felt<br>☐ Impossible<br>→ **Head support:**<br>☐ Satisfactory<br>☐ Possible briefly (<3 seconds)<br>☐ Impossible	
	→ **Firm pressure** *(see Appendix 4)*: Apply a series of firm pressures and relaxations at a steady pace, with open palms on the face = forehead and top of skull, both temples, eye and top of skull, both cheeks, mouth and top of skull<br>Signs of discomfort may include grimacing, attempts to avoid contact, etc.<br>→ **Light touch** *(see Appendix 4)*: With your index finger, stroke different areas of the face, spacing stimulations 5 seconds apart = temples to forehead, cheeks, chin, upper lip, lower lip.<br>Signs of discomfort could include pressing the tongue against the palate.<br>NB: To be taken into account in assessing the sensitivity profile.	→ **Firm pressure:**<br>☐ No reaction<br>☐ Sign of discomfort<br>+ Specify the type of sign:<br>……………………………………………………………………<br>→ **Light touch:**<br>☐ No reaction<br>☐ Sign of discomfort<br>+ Specify the type of sign:<br>……………………………………………………………………	
**4. Mouth opening**	⌚ **Item O1. Initiation of mouth opening:**<br>1. Ask the patient "Open your mouth" 3 times, leaving a minimum of 10 seconds between requests. Level 3 is achieved if the patient opens his/her mouth a minimum of twice.<br>2. If there is no reaction to the request to open the mouth, bring a spoon up to the patient's mouth – repeat 3 times if the patient does not react within 10 seconds.<br>3. If there is no reaction 10 seconds after the third presentation of the spoon, rub the spoon, and then your index finger, back and forth on the lower lip.<br>4. If there is no reaction after 10 seconds, press your thumb against the lower jaw (active assistance).<br>If the patient opens his/her mouth constantly, a greater mouth opening reaction to the approach of the spoon is expected. If there is not this reaction, score 0.<br>**NB**: If the patient does not open his/her mouth with active assistance from the therapist, take this into account in assessing the tonicity profile as "T+ hypercontraction."	☐ *Level 3*: Opens mouth upon command (min 2/3)<br>☐ *Level 2*: Opens mouth upon presentation of spoon<br>☐ *Level 1*: Opens mouth upon lip stimulation<br>☐ *Level 0*: Cannot open mouth or only with active assistance by therapist	Empty spoon<br>Stopwatch
	**Amplitude of mouth opening:** To determine whether food can pass through, verify that the mouth opening (obtained in item O1) equals at least 2 fingers (index and middle fingers)	**Amplitude of mouth opening:**<br>☐ 2 fingers<br>☐ more than 2 fingers<br>☐ less than 2 fingers	

**5. Mouth cavity observations**	Describe the patient's **oral hygiene and dental condition.**	→ **Oral hygiene:**<br>☐ satisfactory ☐ dry ☐ coated<br>→ **Describe teeth:** ☐ Full set of teeth<br>☐ No teeth ☐ Partial set of teeth + specify:<br>→ **Dental condition:**<br>☐ Good ☐ Tartar ☐ Gingivitis ☐ Bleeding<br>☐ Worn ☐ Bruxism ☐ Food residue<br>→ **Dental prosthesis:** ☐ yes ☐ no<br>→ **Appliance for bruxism:** ☐ yes ☐ no<br>→ **Drooling:** ☐ yes ☐ no<br>*If yes:* ☐ left ☐ right	Flashlight
	**Item O2. Endo-buccal secretions:**<br>Observe the presence and amount of saliva or dry or moist mucus secreted in the patient's mouth cavity: cheeks, buccal vestibule, floor of the mouth, tongue and oropharynx. Determine whether the amount of saliva and mucus secreted in the mouth in relation to the size of the mouth cavity is approximately 0%–20%, 20%–80% or 80%–100% or if there are no secretions.<br><br>NB: If the mouth cannot be opened, try to observe secretions at other times (e.g., when the patient yawns). Score 0 if the item cannot be assessed.	☐ *Level 3*: Moist mouth without significant secretions<br>☐ *Level 2*: Few visible secretions (0%–20%)<br>☐ *Level 1*: Moderate amount of secretions (20%–80%)<br>☐ *Level 0*: Substantial amount of secretions (80%–100%)	Flashlight
	Describe the **appearance** of the saliva or mucus secretions in the mouth: texture, color, etc.	**Appearance of secretions:**<br>………………………………………………	
**6. Initiation of saliva swallowing reflex**	**Item P1. Initiation of saliva swallowing reflex:**<br>1. Ask the patient "Swallow your saliva," making an exaggerated head movement and placing your hand on your throat to indicate movement of the larynx. Make this request 3 times, leaving a minimum of 10 seconds between requests. Level 3 is achieved if the patient swallows a minimum of twice.<br>2. If there is no reaction to the request, move on to item "P2. Latency of swallowing reflex triggering upon stimulation." Then score item P1 on the basis of the swallowing upon stimulation observed for item P2, and on the basis of saliva swallowing observed during the examination.	☐ *Level 3*: Swallows saliva upon command (min 2/3)<br>☐ *Level 2*: Swallows saliva spontaneously and upon stimulation<br>☐ *Level 1*: Swallows saliva only upon stimulation<br>☐ *Level 0*: No saliva swallowing spontaneously or upon stimulation	Stopwatch
	If applicable, specify whether the verbal command **triggered incomplete swallowing** (tongue movements or laryngeal raising without triggering of swallowing):	☐ Swallowing started upon verbal command but not completed	

**7. Triggering of saliva swallowing reflex upon stimulation**	🕒 **Item P2. Latency of triggering swallowing reflex upon stimulation:**<br>Brush a cotton swab soaked in a cold, sweet solution across the following areas, 3 times each: (1) tongue apex, (2) base of the tongue, (3) velum (soft palate), and (4) posterior pharyngeal wall.<br>**NB:** If the mouth cannot be opened, stimulate the lips.<br>Record the reaction time after each stimulation. If there is no lip or tongue movement and swallowing does not occur upon stimulation, **wait 10 seconds** before moving on to the next area. If a lip or tongue movement occurs after stimulation, **wait 30 seconds** maximum before moving on to the next area.	☐ *Level 3*: 0 to 5 seconds<br>☐ *Level 2*: 5 to 10 seconds<br>☐ *Level 1*: > 10 seconds<br>☐ *Level 0*: No triggering or not successful	Cotton swab soaked in cold, sweet solution<br><br>Stopwatch
	NB: Stop the stopwatch when the patient triggers a complete swallow (and not merely movements).<br>Note the **time taken** to trigger swallowing in this area in P2. Note which areas triggered swallowing and which ones triggered incomplete swallowing (larynx raised jerkily, start of tongue propulsion, etc.).<br>**Criteria for stopping** scoring the item**:**<br>1. Triggering of a swallowing reflex<br>2. Occurrence of a gag reflex<br>3. No reaction after stimulation of the posterior pharyngeal wall	**Area(s) that triggered swallowing:**<br>☐ Lips ☐ Tongue apex<br>☐ Base of tongue ☐ Velum<br>☐ Posterior pharyngeal wall<br>☐ No swallowing<br><br>**Area(s) that triggered incomplete swallowing:**<br>☐ Lips ☐ Tongue apex<br>☐ Base of tongue ☐ Velum<br>☐ Posterior pharyngeal wall<br>☐ No incomplete swallowing	
	If swallowing is triggered during the stimulations in item P2 but there is no gag reflex, continue the stimulations in P2 to see whether **the gag reflex exists and where it is triggered**.<br><br>NB: To be taken into consideration in the assessment of the sensitivity profile.	**Gag reflex:** ☐ yes ☐ no<br>*If Yes, area(s) that triggered gag reflex:*<br>☐ Lips ☐ Tongue apex<br>☐ Base of tongue ☐ Velum<br>☐ Posterior pharyngeal wall<br><br>*Potential other signs of discomfort:*<br>……………………………………………………………………………	Cotton swab soaked in cold, sweet solution
	If swallowing occurs, note the **quality of laryngeal raising:**	**Laryngeal raising:** ☐ yes ☐ no<br>☐ Abnormal: 0 incomplete 0 slow 0 jerky	

**8. Functional test**	🕓 **Items O3. Lip prehension and O4. Tongue propulsion:**<br>• Prepare a teaspoon containing 5 mL of cold, moderately thick colored solution (IDDSI level 3 texture) (NB: 5 mL = approx. 1 teaspoon).<br>• Restart the initiation of mouth opening protocol (item O1) and present the prepared spoon.<br>• Observe the lip prehension of the spoon (**item O3**). If the patient does not react, stimulate him/her verbally by saying "Take the spoon."<br>• If there is no mouth opening or lip prehension upon presentation of the spoon but it is possible to open the mouth passively, place the contents of the spoon on the patient's tongue and observe the reactions.<br>• Start the stopwatch when lip prehension occurs or the liquid is placed on the tongue.<br>• Observe the tongue propulsion (**item O4**), placing one hand under the floor of the patient's mouth to feel any tongue movements.<br>• Check for the existence of any post-swallowing oral stasis.<br><br>If the patient does not present any lip or tongue reaction **after 10 seconds**, score 0.<br>If the patient presents complete lip prehension (level 3 for item O3), do two more trials with an empty spoon to verify the stability of prehension. If the patient continues to succeed at this item, score level 3; if the patient fails the second or third time, score level 2.<br><br>**NOTE: If the patient does not show any saliva swallowing upon stimulation (level 0 for item P1), no spontaneous swallowing is observed or the patient has a strong bite reflex or trismus, the functional test should not be carried out.** In this case, "tongue propulsion" should be scored 0 and "lip prehension" should be tested with an empty spoon.<br><br>**NB:** Similarly, if the functional test cannot be done because the mouth will not open, the "tongue propulsion" and "lip prehension" items must be scored 0.	**O3. Lip prehension:**<br>◻ *Level 3*: Consistently correct, spontaneous lip prehension<br>◻ *Level 2*: Appropriate lip prehension but not consistently or only upon verbal stimulation<br>◻ Level 1: Incomplete lip prehension spontaneously or upon verbal stimulation<br>◻ *Level 0*: No lip prehension (no reaction or tightening of lips)<br><br>**O4. Tongue propulsion:**<br>◻ *Level 3*: Appropriate tongue propulsion<br>◻ *Level 2*: Pathological tongue propulsion, possibly with post-swallowing stasis<br>◻ *Level 1*: A few tongue movements but not sufficient to propel the bolus<br>◻ *Level 0:* No tongue movement: passive transfer of the bolus to the pharyngeal level, stagnation in mouth or expulsion when drooling	Stopwatch<br><br>Teaspoon<br>5 mL of cold, colored thickened solution with IDDSI level 3 texture (moderately thick)

	In addition to the lip prehension and tongue propulsion items above, note whether there is any **triggering of swallowing and/or coughing and/or going down the wrong way (inhalation).**	→ **Triggering of swallowing reflex:**<br>◻ yes ◻ no<br>→ **Triggering time after the liquid has touched the tongue:**<br>… sec<br>→ **Multiple swallows:** ◻ yes ◻ no<br>→ **Coughing episode:** ◻ yes ◻ no<br>*If yes:* ◻ before swallowing ◻ after swallowing ◻ remotely<br>→ **Signs of increased congestion or dyspnea:**<br>◻ yes ◻ no<br>→ **Other signs of inhalation:** ………………..........................	
	Note the characteristics of triggering of **tongue movements:**	**Spontaneous tongue movements:**<br>→ *Not associated with attempts to swallow*: ◻ yes ◻ no<br>→ *During attempts to swallow*<br>saliva: ◻ yes ◻ no<br>→ *In response to stimulation of the face, lips or tongue itself:*<br>◻ yes ◻ no<br>+ *If yes, specify the stimulation that triggered tongue movements:*<br>…………………………………..	
	Note signs of any **primitive reflexes** upon mouth opening and lip prehension:	→ **Bite reflex:** ◻ yes ◻ no<br>→ **Sucking reflex:** ◻ yes ◻ no<br>→ **Chewing reflex:** ◻ yes ◻ no	
	In case of swallowing, note the **quality of laryngeal raising.**	**Laryngeal raising:**<br>◻ yes ◻ no<br>◻ Abnormal: O incomplete O slow O jerky	
	**Item P3. Tracheostomy:**<br>Question the care team, look at the patient's file and/or observe the patient's tracheostomy.	◻ *Level 3*: Tracheostomy with ongoing weaning – tube can be plugged with a finger – or no tracheostomy<br>◻ *Level 2*: Tracheostomy without a cuff or with a permanently deflated cuff<br>◻ *Level 1*: Tracheostomy with a cuff, ongoing deflation<br>◻ *Level 0*: Tracheostomy with an inflated cuff<br>*If there is no tracheostomy*, did the patient have one in the past?<br>◻ yes ◻ no	Patient's file

9. Respiration	**Item P4. Respiration and upper and lower airway congestion:**<br>Question the care team, look at the patient's file and/or observe the patient.<br><br>To assess the level of congestion in the upper and lower airways, look for the **severity of the following signs of congestion** in the patient: noisy or whistling breathing, coughing that indicates the presence of mucus, etc. It is also important to consider the **number of intraoral or endotracheal aspirations** done by the care team or the patient himself/herself. Finally, the need for **respiratory kinesiotherapy treatment** should be considered.	☐ *Level 3*: no congestion<br>☐ *Level 2*: little congestion<br>☐ *Level 1*: moderate congestion<br>☐ *Level 0*: frequent bronchopneumonia or heavy congestion	Patient's file
	If **congestion** exists, specify the type.	→ **Breathing aid systems:**<br>☐ humidifiers ☐ oxygen<br>In case of oxygen treatment, number of liters or %:<br>…………………<br>→ **Upper airway congestion:** ☐ yes ☐ no – specify:<br>……………………………………………………………………………<br>→ **Lower airway congestion:** ☐ yes ☐ no – specify:<br>……………………………………………………………………………<br>→ **Frequency of aspirations:**<br>……………………………………………………………………………<br>→ **Frequency of respiratory kinesiotherapy:**<br>……………………………………………………………………………	
10. Voice Articulation Language	Note the ability to produce **spontaneous sounds, articulate words or reliable communication** (according to the SECONDs, correct responses to 5 simple closed-ended autobiographical questions):	→ **Spontaneous noises/sounds:** ☐ yes ☐ no<br>→ **Articulate words:** ☐ yes ☐ no<br>→ **Reliable communication:** ☐ yes ☐ no	
11. Tonicity and sensitivity profiles	**Tonicity profile**:<br>Observe the patient's facial appearance at rest, during tactile stimulations and during initiation of mouth opening tests.	☐ *T– profile: hypotonicity*: mouth continuously open, drooling<br>☐ *T+ profile: hypertonicity/tightness*: clenches teeth continuously and/or during tactile stimulation, resists mouth opening<br>☐ *T neutral profile*: no signs of hypotonicity or hypertonicity	
	**Sensitivity profile**:<br>Observe reactions to external and internal stimulation in the orofacial area and the gag reflex.	☐ *S– profile: hyposensitivity*: no reaction to tactile stimulation, weak gag reflex or none<br>☐ *S+ profile: hypersensitivity*: startles, grimaces during light touch tactile stimulation, exaggerated gag reflex<br>☐ *S neutral profile*: no signs of hyposensitivity or hypersensitivity	

**SWADOC-scored – Total : /24**	**SWADOC-scored – oral phase: /12**<br><br>**SWADOC-scored – pharyngeal phase: /12**	**Tonicity profile: T+ / Tn / T–**<br><br>**Sensitivity profile: S+ / Sn / S–**
**Comments:**		

APPENDICES

Appendix 1: items of the *Food Intake Level Scale* (FILS)[2]

No oral intake
Level 1: No swallowing training is performed except for oral care.
Level 2: Swallowing training not using food is performed.
Level 3: Swallowing training using a small quantity of food is performed.
Oral intake and alternative nutrition
Level 4: Easy-to-swallow food less than the quantity of a meal (enjoyment level) is ingested orally.
Level 5: Easy-to-swallow food is orally ingested in one to two meals, but alternative nutrition is also given.
Level 6: The patient is supported primarily by ingestion of easy-to-swallow food in three meals, but alternative nutrition is used as a complement.
Oral intake alone
Level 7: Easy-to-swallow food is orally ingested in three meals. No alternative nutrition is given.
Level 8: The patient eats three meals by excluding food that is particularly difficult to swallow.
Level 9: There is no dietary restriction, and the patient ingests three meals orally, but medical considerations are given.
Level 10: There is no dietary restriction, and the patient ingests three meals orally (normal).

Comments:

- Swallowing training: Training conducted by an expert, well-instructed caregiver, or the patient himself/herself to improve the swallowing function.
- Easy-to-swallow food: Food that is prepared so that it is easy to swallow even without mastication, for example, meat and vegetables are gelatinized or homogenized in a mixer.
- Alternative nutrition: Non-oral nutrition such as tube feeding and drip infusion.
- Food that is particularly difficult to eat: dry and brittle food, hard food, water, and so on.
- Medical considerations: guidance, tests, examinations, and so on, for symptoms suggestive of swallowing disorders such as choking and the feeling of food remaining in the pharynx.

Appendix 2: International Dysphagia Diet Standardisation Initiative (IDDSI)[1]

For the corresponding FILS scores, note the textures of food and drinks offered to patients using the IDDSI terminology.

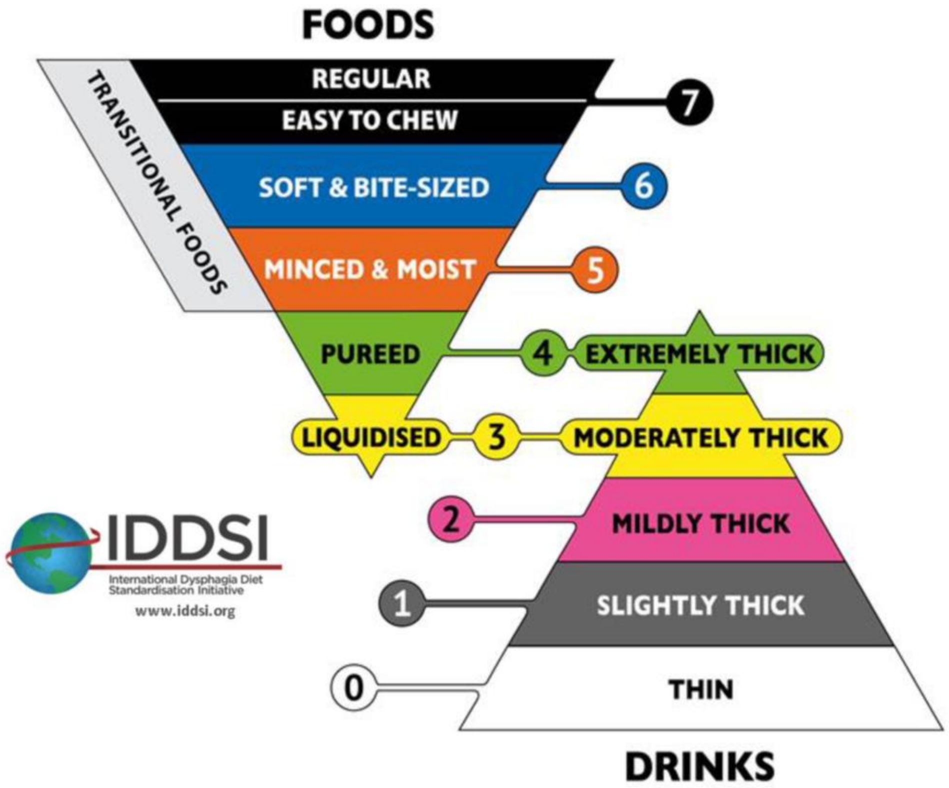

© The International Dysphagia Diet Standardisation Initiative 2019
@https://iddsi.org/framework.

Appendix 3: arousal protocol of the Coma Recovery Scale Revised (CRS-R) [5,6]

AROUSAL FACILITATION PROTOCOL (AFP) ©2004

GUIDELINES

1) The goal of this intervention is to prolong the length of time the patient maintains arousal (i.e. eye opening)

2) The protocol is administered any time the patient is observed to:

   - Exhibit sustained eyelid closure **AND/OR**

   - Stops following commands for a period of at least one minute

3) Readminister the arousal facilitation protocol when

   - Sustained eye closure re-occurs **OR**

   - Behavioral responsiveness ceases despite sustained eye opening

INTERVENTIONS

Deep Pressure:

1) Present deep pressure stimulation unilaterally to the face, neck, shoulder, and sternocleidomastoid muscles. The muscle should be firmly grasped at its base between the thumb and forefinger. While squeezing the muscle firmly, it should be "rolled" back and forth through the fingertips three to four times. This procedure should be repeated sequentially working from the facial musculature to the sternocleidomastoid. The examiner should assure that there are no intravenous lines, local injuries (e.g. fractures, contusions, decubiti) or systemic complications (e.g. heterotopic ossification) before administering deep pressure.

2) Administer same on contralateral side.

Appendix 4: External stimulations of the orofacial area

Objective:
This subtest assesses the patient's reactions to facial tactile stimulation. It is mainly intended to determine the patient's sensitivity and tonicity "profiles" in reaction to stimulations in order to guide the patient's treatment.
This subtest is inspired by the orofacial sensitivity subtest of the Comprehensive Assessment Measure for the Minimally Responsive Individual (CAMMRI)[7] in the case of light touch and by the stimulation methods for sensory processing disorder (sensory dysorality) proposed by Catherine Senez[8] for firm pressure ("going around the house").

Materials:
- Latex/vinyl gloves

Test administration:
Tell the patient you are going to touch different areas of his/her face.
Follow the specific order in the data form for the areas targeted. First do the firm pressure test, followed by the light touch test.

1) Firm pressure

Using both hands, with the palms open and placed on the patient's head, engage in a series of sequences of contact, pressure, relaxation of pressure and removal of hands. The pressures must succeed each other at a steady pace, and very quickly. The pressure applied should be moderate to firm in one location in the area to be stimulated (do not stroke the whole area).

Description of the stimulation:
- Forehead and top of skull
- Both temples
- One eye and top of skull (cup your hand to avoid touching the eye)
- The other eye and top of skull
- Both cheeks
- Mouth and top of skull

2) Light touch

During light stimulation, stroke with your index finger. Touch each area only once. Space the simulations 5 seconds apart.

**Areas to stimulate**	**Description of the stimulation**
a) Temples/ forehead	Stroke continuously from the temple to the midline of the face, on one side and then the other
b) Cheeks	Start at the height of the middle of the ear, follow the cheekbone and stop in the mid-eye area
c) Chin	With your index finger flattened, move up the length of the chin
d) Upper lip	Stroke the lip from left to right
e) Lower lip	Stroke the lip from left to right

References

1. Cichero JAY, Lam P, Steele CM, et al. Development of international terminology and definitions for texture-modified foods and thickened fluids used in dysphagia management: The IDDSI framework. *Dysphagia*. 2017;32(2):293-314. doi:10.1007/s00455-016-9758-y

2. Kunieda K, Ohno T, Fujishima I, Hojo K, Morita T. Reliability and validity of a tool to measure the severity of dysphagia: The Food Intake LEVEL Scale. *Journal of Pain and Symptom Management*. 2013;46(2):201-206. doi:10.1016/j.jpainsymman.2012.07.020

3. Aubinet C, Cassol H, Bodart O, et al. Simplified Evaluation of CONsciousness Disorders (SECONDs) : A new tool to assess consciousness in severely brain-injured patients. *Annals of Physical and Rehabilitation Medicine*. Published online 2020. doi:10.1016/j.rehab.2020.09.001

4. Sanz LRD, Aubinet C, Cassol H, et al. SECONDs administration guidelines: A fast tool for assessing consciousness in brain-injured patients. *Journal of Visualized Experiments*. 2020;In press.

5. Giacino JT, Kalmar K, Whyte J. The JFK Coma Recovery Scale – Revised: Measurement characteristics and diagnostic utility. *Archives of Physical Medicine and Rehabilitation*. 2004;85(12):2020-2029. doi:10.1016/j.apmr.2004.02.033

6. Bodien YG, Chatelle C, Giacino JT. Arousal protocol of the Coma Recovery Scale – Revised. Accessed January 12, 2021. https://www.sralab.org/rehabilitation-measures/coma-recovery-scale-revised

7. Gollega A, Meghji C, Renton S, et al. Multidisciplinary assessment measure for individuals with disorders of consciousness. *Brain Injury*. 2015;29(12):1460-1466. doi:10.3109/02699052.2015.1071426

8. Senez C. *Rééducation des troubles de l'oralité et de la déglutition*. 2nd ed. De Boeck Supérieur; 2015.
