# Supplementary material for: The Development and Validation of the SWADOC: A Study Protocol for a Multicenter Prospective Cohort Study
Source: Front Neurol. 2021 Apr 29;12:662634. doi: 10.3389/fneur.2021.662634 (PMC8116670; doi:10.3389/fneur.2021.662634)
Supplement: Supplementary file 3 [file Presentation_3.pdf]

Supplementary Material 3. SWADOC-scored (French version)

	Items	Niveau 0	Niveau 1	Niveau 2	Niveau 3
**Phase orale**	**1. Initiation d'ouverture buccale**	◻ Ouverture de la bouche impossible ou seulement sur aide active du thérapeute	◻ Ouverture buccale sur stimulation labiale	◻ Ouverture buccale à l'approche de la cuillère	◻ Ouverture buccale sur commande (min 2/3)
	**2. Sécrétions endo-buccales**	◻ Sécrétions en quantité significative (80-100%)	◻ Sécrétions en quantité modérée (20-80%)	◻ Peu de sécrétions (0-20%)	◻ Bouche humide mais sans sécrétions significatives
	**3. Préhension labiale**	◻ Aucune préhension labiale (pas de réaction ou serrage des lèvres)	◻ Préhension labiale incomplète en spontané ou sur stimulation verbale	◻ Préhension labiale adéquate mais non-systématique ou uniquement sur stimulation verbale	◻ Préhension labiale correcte et spontanée systématiquement
	**4. Propulsion linguale**	◻ Aucun mouvement lingual : passage passif du bolus au niveau pharyngé, stagne en bouche ou ressort en bavage	◻ Quelques mouvements linguaux mais insuffisants pour propulser le bolus	◻ Propulsion linguale pathologique avec présence possible de stases post-déglutition	◻ Propulsion linguale adéquate
**Phase pharyngée**	**1. Initiation du réflexe de déglutition salivaire**	◻ Pas de déglutition de salive que ce soit sur stimulation ou spontanément	◻ Déglutition de salive uniquement sur stimulation	◻ Déglutition de salive spontanée et sur stimulation	◻ Déglutition de salive sur commande (min 2/3)
	**2. Latence de déclenchement du réflexe de déglutition sur stimulation**	◻ Pas de déclenchement ou non-réalisable	◻ > 10 sec	◻ Entre 5 et 10 secondes	◻ Entre 0 et 5 secondes
	**3. Trachéotomie**	◻ Trachéotomie avec ballonnet gonflé	◻ Trachéotomie avec ballonnet en cours de sevrage	◻ Trachéotomie sans ballonnet ou avec ballonnet dégonflé en permanence	◻ Trachéotomie en cours de sevrage, ou absente
	**4. Respiration et encombrement haut et bas**	◻ Bronchopneumonies fréquentes ou encombrement important	◻ Encombrement modéré	◻ Peu d'encombrement	◻ Pas d'encombrement
**SWADOC-scored phase orale: /12**		**SWADOC-scored phase pharyngée: /12**		**SWADOC-scored total: /24**	
