# Supplementary material for: The Development and Validation of the SWADOC: A Study Protocol for a Multicenter Prospective Cohort Study
Source: Front Neurol. 2021 Apr 29;12:662634. doi: 10.3389/fneur.2021.662634 (PMC8116670; doi:10.3389/fneur.2021.662634)
Supplement: Supplementary file 4 [file Presentation_4.pdf]

Supplementary Material 4. The FOTT-SAS (table adapted from Mortensen et al., 2016)

Items	Yes	No
1) Conscious and/or respond to verbal address?		
2) Abel to sit upright with some degree of head control?		
3) Oral transport of saliva?		
4) Spontaneous or facilitated swallowing of saliva?		
5) Coughing following swallowing of saliva?		
6) Gurgling breath sound following swallowing of saliva?		
7) Difficulties in breathing following swallowing of saliva?		
Based on the above questions, should oral intake be initiated?<br>(Oral intake should be initiated if items 1-4=Yes and items 5-7=No)		
